# Heterocyclizations at the Isocyanide Carbon: Mechanistic Insights into the 2‑Isocyanoaniline to Benzimidazole Conversion

**DOI:** 10.1021/acs.joc.5c02202

**Published:** 2026-01-15

**Authors:** Mateo Alajarin, Marta Marin-Luna

**Affiliations:** Departamento de Química Orgánica, Facultad de Química, Regional Campus of International Excellence “Campus Mare Nostrum”, 16751 Universidad de Murcia, E-30100 Murcia, Spain

## Abstract

Benzo-fused heteroazacycles can form through spontaneous cyclizations of phenylisocyanides bearing some electron donor substituents at the *ortho* position. Although such reactions have been known since the 1970s, a clear mechanistic understanding of these transformations remains absent. Here, we disclose a detailed computational analysis aimed at elucidating the more plausible mechanism for one of these cyclizations, the *o*-isocyanoaniline to benzimidazole conversion. Our results show that a transient 1,4-diazabutatriene could play a key role in this cyclization by enabling a reaction pathway of lower barrier than the unimolecular concerted process.

## Graphical abstract

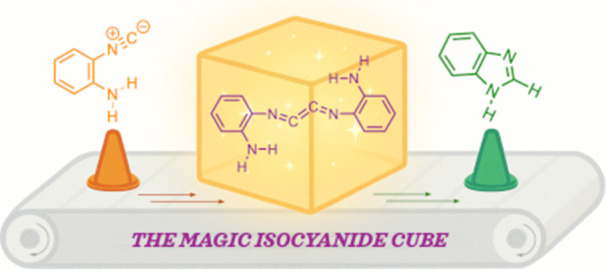

Isocyanides (isonitriles) are versatile functional groups that take part in multiple organic transformations either as nucleophiles or electrophiles.^1^ Due to such remarkable dual reactivity, isocyanides have come to be known as chameleonic compounds.^2–5^ Among other things, isocyanides are capable of forming complex azacyclic compounds via multicomponent reactions.^6^ In this sense, the formation of benzo-fused heterocycles **2**, starting from *ortho*-substituted phenylisocyanide fragments **1** (X = O, NH), is an interesting reported protocol (Scheme 1). *o*-Isocyanophenol evolves to benzoxazole upon thermal treatment,^7–11^ and its amino partner has been described as an unstable species due to its rapid evolution to benzimidazole.^12^ It is noteworthy that the success of these transformations is contingent upon the direct bonding of the heteroatom to the aromatic ring. In contrast, *o*-(hydroxymethyl)­phenyl isocyanide exhibits stability under thermal conditions.^13^ Reverse reactions, from benzo-fused azacycles toward *o*-substituted phenylisocyanides, have also been reported, proceeding under photochemical activation.^8,10^ Curiously, while the conversion of benzothiazole into *o*-isocyanobenzenethiol is a known reaction, there is no experimental evidence substantiating the inverse transformation, from isocyano-thiol to benzothiazole.^13^

**Scheme 1:**
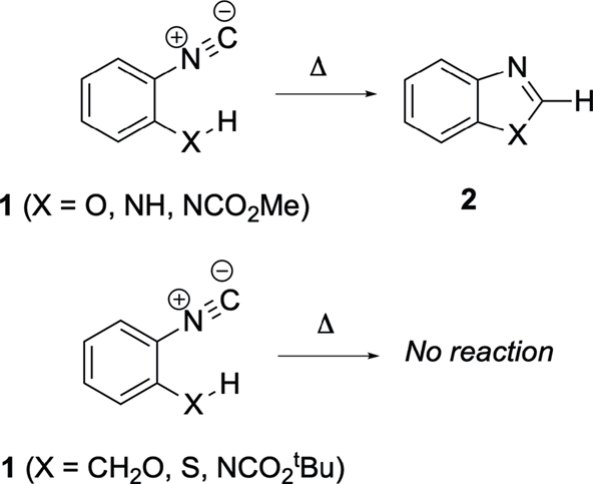
Attempted Reported Cyclizations of *ortho-*Substituted Phenylisocyanides **1**, whether Successful or Not

Other types of phenylisocyanides *ortho*-decorated with *N*-acyl functions seem to exhibit analogous transformations. While *tert*-butyl (2-isocyanophenyl)­carbamate has been reported to be stable under thermal reaction conditions,^14^ other 2-isocyanophenyl carbamates undergo, when warmed up, an intramolecular cyclization to give the corresponding 1-acylbenzimidazoles (Scheme 1).^15–17^

While these are recognized transformations, to our knowledge, their respective mechanisms have not been clarified. Our recent research in the chemistry of azido-substituted 2-(heteroaryl)­phenyl isocyanides^18,19^ has led us to focus our attention on the cyclizations summarized in Scheme 1 and their intriguing mechanisms. Herein, we disclose the results of our computational scrutiny of such mechanisms at the PCM­(CHCl_3_)/M06-2x/aug-CC-pvTZ//M06-2x/aug-CC-pvDZ theoretical level, concluding with the probable participation of 1,4-diazabutatrienic dimers of the starting *ortho*-substituted phenylisocyanides.

We first proposed the two-step process starting with the formation of an X–C bond in **1**, leading to the heterocyclic ylide **3**, followed by a 1,2-H shift, where H migrates as a proton, from the XH fragment to the carbanionic C atom, finally giving rise to the benzoheterocycle **2**. However, all attempts to locate either the transition structures corresponding to the cyclization first step or the cyclized ylide **3** failed, including those incorporating explicit solvent molecules in the calculations (Scheme 2). On the other hand, Reva et al. proposed that the transformation of 2-isocyanophenol **1** (X = O) into benzoxazole occurs by an alternative two-step mechanism, involving the initial 1,5-H shift from the OH group toward the C atom of the isocyanide at **1**, leading to the conjugated nitrile ylide **4** (X = O) that further collapses into the respective **2** (Scheme 2).^8^ Curiously, these authors apparently neither computed the first step, the 1,5-H shift, nor showed the transition structure of the second step, the respective final cyclization of **4** to **2**. They present only a relaxed PES-scan of this latter step as the only support of their mechanistic proposal. Probably, they could not locate the two transition structures of this two-step process. Indeed, this was also our case after many unsuccessful attempts.

**Scheme 2:**
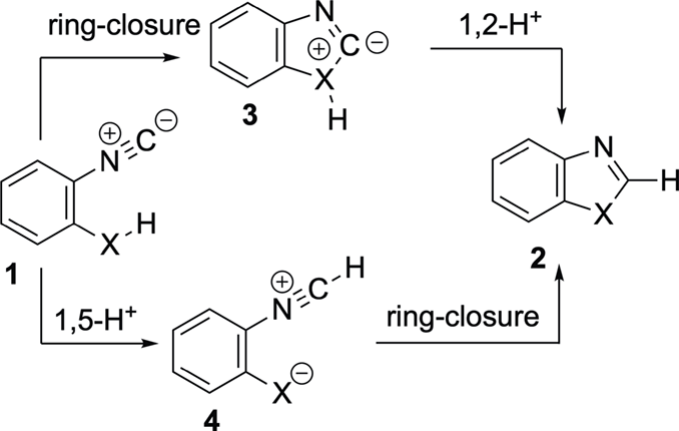
Mechanistic Proposals for the Transformation of Isocyanides **1** into Benzo-Fused Heteroazacycles **2**

Notwithstanding, our calculations show that the three scrutinized **1** to **2** cyclizations (X = O, NH, NCO_2_Me) could occur in a concerted polar manner (Figure 1a). Our DFT calculations predict that the amino-isocyanide must pay an energy penalty of 58.9 kcal mol^–1^ to evolve toward benzimidazole **2**. In the case of carbamate, the computed free energy barrier is 54.1 kcal mol^–1^. The lowest, although still high, computed free energy barrier is associated with the formation of benzoxazole (47.5 kcal mol^–1^). These concerted processes were ascertained by the intrinsic reaction coordinate analyses performed on the located transition structures **TS**_**conc**_ (Figure 1b for model X = NH). The respective single steps start by the rehybridization of the sp-N atom of the isocyanide moiety to sp^2^, thus placing the C atom well oriented to abstract the H atom of the XH group (**A** in Figure 1b) while moving toward the transition structure **TS**_**conc**_. In the second part of the reaction coordinate, the residual NH group is then capable of linking to the C atom of the isocyanide, thus forming the final five-membered ring (**B** in Figure 1b). With these results in hand, we do not consider these concerted mechanisms as plausible because the computed high activation energies seem not compatible with the reported smooth reaction conditions (room temperature).

**Figure 1:**
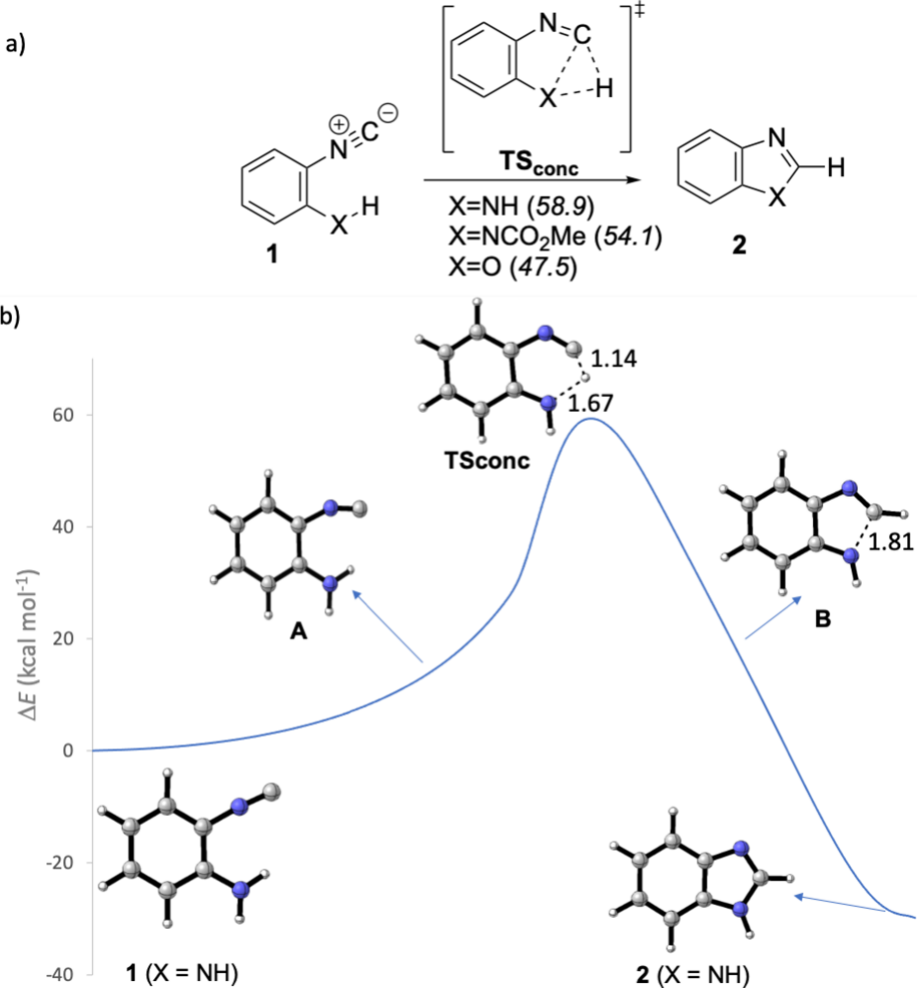
(a) Computed concerted mechanism for the transformation of derivates **1** into benzoazacycles **2**. Activation energies of the respective **TS_conc_** structures are shown in kcal·mol^–1^. (b) Computed energy profile along the reaction coordinate for the transformation of *o*-isocyanoaniline 1 (X = NH) into benzimidazole **2** involving **TS_conc_**. Distances are given in Angstroms (Å).

After discarding the three unimolecular mechanisms above, we next tested some bimolecular scenarios, as a transformation mediated by a second isocyanide molecule acting as a kind of catalyst in a total concerted mechanism (see the Supporting Information). Unfortunately, we cannot validate that proposal since the key transition structure was not located.

The experimental evidence that bulky (2-isocyanophenyl) carbamates are stable under thermal conditions led us to consider if the **1 → 2** conversion is due to the delocalization of the X atom lone pair on the aromatic system, rather than to the acidity of the XH proton or the nucleophilic character of X.

For this reason, and following a logical approach, we next gave a chance to see a stepwise version of the bimolecular mechanistic proposal commented above. We conceived such process as initiated by the formation of a dimer of the *ortho*-HX-phenyl isocyanide molecules. In 1965, Ugi et al. already proposed that phenylisocyanide is in equilibrium with the dimer formed by linking the isocyano carbon atoms of two molecules by means of a double bond.^20^ In fact, such dimers, the elusive 1,4-diazabutatrienes, have been envisaged as intervening in multiple transformations of isocyanides although they have been never isolated.^21^

Taking as computational model **1** (X = NH) and admitting its initial dimerization to 1,4-diazabutatriene **INT1** (Scheme 3), two routes emerge henceforth depending on how the carbon atoms of the NCCN fragment act either as nucleophilic, by the transfer of one proton of the amino group to the C atom, or as electrophilic, by the attack of the amino group on the C atom. Afterward, each route could branch off into secondary pathways toward benzimidazole **2**. All of these routes have been computed (see the Supporting Information), and we will show here only our results on the most favorable pathways in energy terms (Scheme 3).

**Scheme 3:**
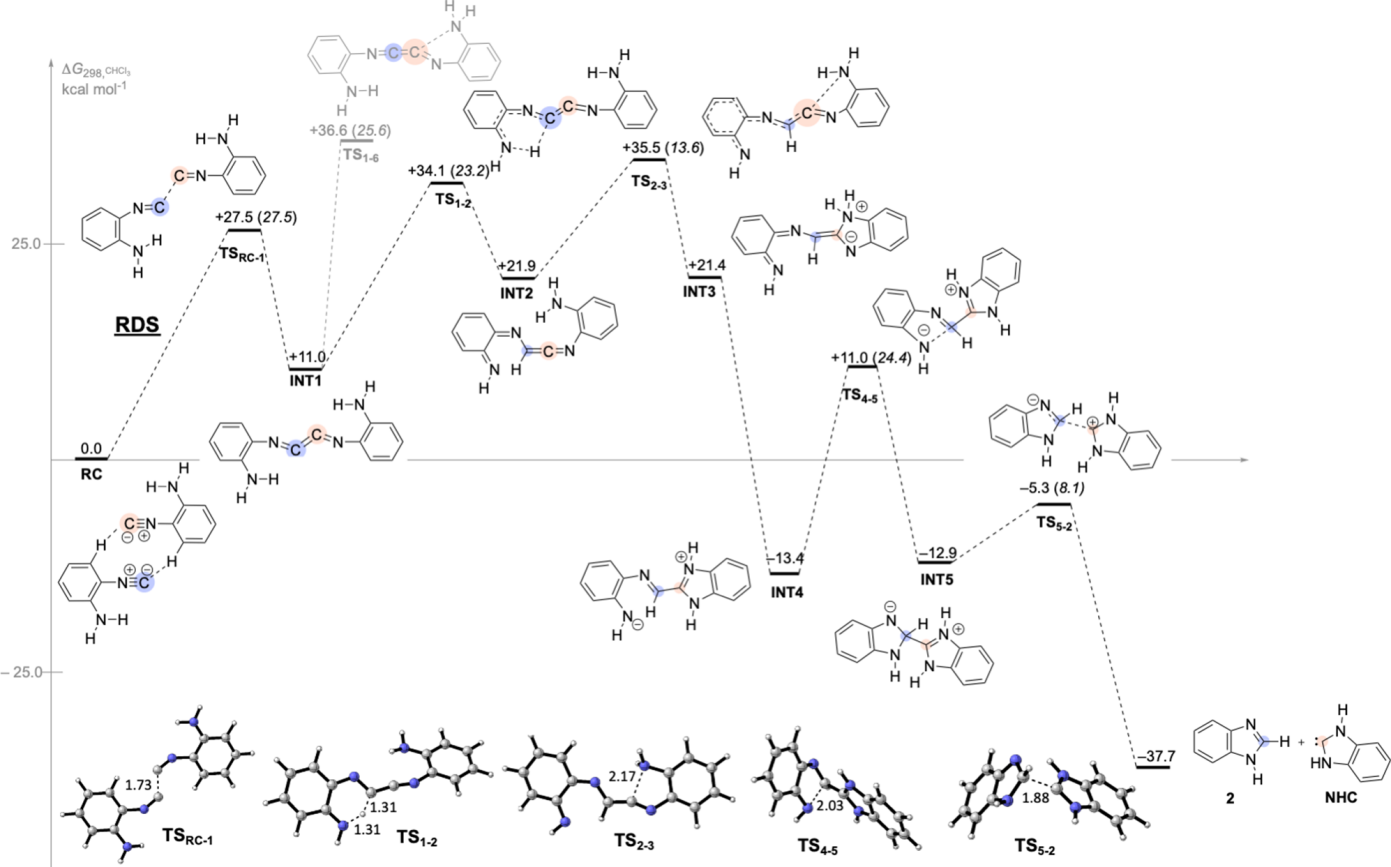
Computed Mechanism for the Formation of Benzimidazole **2** via the 1,4-Diazabutatriene Intermediate **INT1** ^a^

a 3D structures of key transition structures are shown. Energies are given in kcal mol^–1^, while the distances are given in Angstroms (Å).

Two molecules of *o*-aminophenyl isocyanide interact with each other to form a reactant complex **RC**,^22^ whose structural organization provides an optimal scenario to a successful head-to-head dimerization process, forming the 1,4-diazabutatriene intermediate **INT1** (Δ*G* ^‡^ **TS**_**RC‑1**_ = 27.5 kcal mol^–1^). This dimerization step is computed as the rate-determining step (RDS) of the entire process. This step was also explored on 2-isocyanophenol **1** (X = O) and on the carbamate derivative **1** (X = NCO_2_Me). Pleasantly, our calculations predict that the activation energies of both processes are lower than those of the previously computed concerted mechanisms: 26.3 and 27.5 kcal mol^–1^, respectively.

Next, **INT1** could experiment the nucleophilic attack of the amino N atom to the nearest cumulated carbon by surpassing an energy barrier of 25.6 kcal mol^–1^ via **TS**_**1–6**_. However, we found a lower energetic pathway with a barrier of 23.2 kcal mol^–1^, converting **INT1** into the ketenimine intermediate **INT2**, via **TS**_**1–2**_, by a 1,5-H transfer from the amino group to the nearest cumulated C atom. The difference between the activation barriers of the latter two processes could be reasonably interpreted. In the case of the nucleophilic attack of the amino N atom, the structural distortion needed to reach the cumulated carbon atom is notable, whereas in the 1,5-H step, the migrating H atom is already in the vicinity of the recipient C atom. After that, the ketenimine fragment of **INT2** is prone to experiment the classical nucleophilic attack of the pendant amino group to its central carbon atom (Δ*G* ^‡^ **TS**_**2–3**_ = 13.6 kcal mol^–1^), yielding **INT3**, in a proton-transfer equilibrium with **INT4** (−13.4 kcal mol^–1^). Note here the low barrier of the nucleophilic addition leading to **INT3** compared to that surpassing **TS**_**1–6**_. This fact might be a consequence of the unequivocal electrophilic character of the central C atom of the ketenimine function, similar to that of **INT2**. A subsequent ring closure at **INT4** via **TS**_**4–5**_ (Δ*G* ^‡^ **TS**_**4–5**_ = 24.4 kcal mol^–1^) affords the zwitterionic structure **INT5**. The low-energy rupture of the exocyclic C–C bond results in the formation of benzimidazole **2** and the carbene **NHC**.^23^

The last step of our mechanistic proposal consists of the transformation of **NHC** into its more stable isomer benzimidazole **2** (Scheme 4). Although the much higher stability of **2** in comparison with **NHC** has been disclosed in advance by Alkorta and Elguero,^24^ to our knowledge, the precise mechanism of the conversion of **NHC** into **2** has not been computed until now. We have found that the intramolecular 1,2-H transfer via **TS**_**a**_ is nearly 35 kcal mol^–1^ more energetically costly than the bimolecular version through the 6-center transition structure **TS**_**b**_ (Scheme 4). These findings agree with those described for other N-heterocyclic carbenes.^25^

**Scheme 4:**
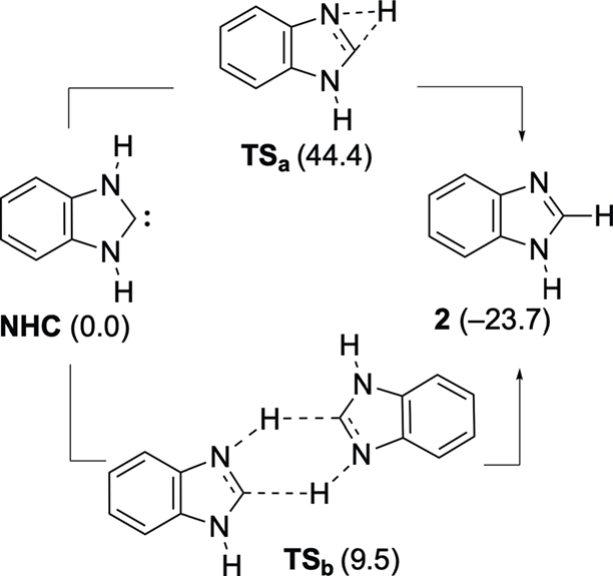
Computed Alternative Transformations of NHC into **2** ^a^

a Relative free Gibbs energies are given in kcal mol^–1^.

In summary, herein, we disclose a detailed computational study on the transformation of *o*-isocyanoaniline into benzimidazole, a transformation briefly described in the bibliography but not mechanistically scrutinized up to now. Multiple reaction pathways have been computed, and the results show that the most favorable one involves the dimerization of the reactant to form a 1,4-diazabutatriene intermediate. This step continues with a proton transfer from the amino group to one of the cumulated carbon atoms of that intermediate, followed by two cyclizations and the dissociation into two molecules of benzimidazole. This mechanism has been computed to be more favorable, in energy terms, than the unimolecular one previously proposed, which we computed to occur by a concerted mechanism with a high energy barrier.

A relevant conclusion of this work is that mechanistic pathways involving the transient formation of dimeric 1,4-diazabutatrienes should be taken into consideration in the study of the mechanism of other reported cyclizations involving the carbon atom of isocyanide functions. This could be the case for the Van Leusen reaction and related 5-endo-dig cyclizations. Computational insights on this theme are currently underway in our research group.

## Supplementary Material



## Data Availability

The data underlying this study are available in the published article and its Supporting Information.

## Notes

Computational data and Cartesian coordinates (PDF)
