# Supplementary material for: Heterocyclizations at the Isocyanide Carbon: Mechanistic Insights into the 2‑Isocyanoaniline to Benzimidazole Conversion
Source: J Org Chem. 2026 Jan 15;91(4):1837–41. doi: 10.1021/acs.joc.5c02202 (PMC12865753; doi:10.1021/acs.joc.5c02202)
Supplement: Supplementary file 1 [file jo5c02202_si_001.pdf]

Supporting Information

Heterocyclizations at the Isocyanide Carbon: Mechanistic Insights into the 2-Isocyanoaniline to Benzimidazole Conversion

Mateo Alajarin, Marta Marin-Luna

Departamento de Química Orgánica, Facultad de Química, Regional Campus of International Excellence "Campus Mare Nostrum", Universidad de Murcia, E-30100, Murcia, Spain

1. Computational Study* *S2

1.1. Computational Methods S2

1.2. Computational Benchmark S2

1.3. Computed Mechanisms S2

1.4. IRC calculations S4

1.5. Computational data S5

1.6. Cartesian Coordinates S6

2. Bibliography* *S16

1. Computational Study

1.1. Computational Methods

Geometries of the molecules were optimized by using the M06-2x functional[1] with the aug-CC-pvDZ basis sets.[2] The method has been used in related isocyanide works.[3]The nature of minima and transition structures of all found stationary points on the potential energy surface was confirmed by frequency analysis at the same level of theory. The stability of the resulting wavefunctions were checked for all the optimized structures.[4] IRC calculations were performed on the optimized transition structures to confirm that each of them indeed connected the indented minima intermediates. The computed thermochemical corrections (G_{corr}) at M06-2x/aug-CC-pvDZ level were added to the electronic energy calculated at the PCM($CHCl_3$)/M06-2X/aug-CC-pvTZ[2] level to yield corrected Gibbs free energies *G* at 298.15 K ($G_{298,sol}$). Chloroform was chosen as model solvent because it has been reported as efficient in cyclization reactions of isocyanides.[5] All the calculations were performed by means of Gaussian 16 C. 01 software[6] and the ultrafine grid implemented in that program was applied. 3D structures were plotted by using the CYLView software.[7]

1.2. Computational Benchmark

We validated our computational results by performing single-point energy calculations at various high theoretical level on some relevant optimized structures at the M062x/aug-CC-pvDZ level. We selected four different DFT hybrid-functionals: PBE0-D3, BMK, wB97xD and M062X. In all cases, we used the aug-cc-pVTZ as basis set and PCM($CHCl_3$) as solvation model (Table S1).

Table S1. Benchmark analysis. Relative Gibbs free energies (ΔG, **1** (X=NH) was taking as reference) and barrier energies of transition structures ($\Delta G^{\neq}$) are shown in kcal mol^{-1}.

	**PBE0-D3**		**BMK**		**wB97XD**		**M06-2X**	
	**ΔG**	**$\Delta G^{\neq}$**	**ΔG**	**$\Delta G^{\neq}$**	**ΔG**	**$\Delta G^{\neq}$**	**ΔG**	**$\Delta G^{\neq}$**
**1 (X=NH)**	0		0		0		0	
**TS_{conc} (X=NH)**	51.0	51.0	58.8	58.8	56.8	56.8	58.9	58.9
**RC**	7.8		10.2		8.3		8.6	
**TS_{RC-1}**	26.8	19.0	36.8	26.6	33.2	25.0	36.1	27.5
**INT1**	4.8		24.1		16.8		19.6	
**TS_{1-2}**	25.4	20.6	44.5	20.4	39.4	22.6	42.7	23.2
**TS_{1-7}**	30.3	25.5	52.2	28.1	41.7	24.9	45.5	26.0
**INT5**	-21.8		-5.2		-9.5		-4.8	
**TS_{5-6}**	1.7	23.5	22.3	27.4	13.9	23.4	19.6	24.4

1.3. Computed Mechanisms

We tested some bimolecular scenarios, as the one summarized in Scheme S1. This mechanistic sequence contemplates the temporal linking of two units of isocyanide by formation of a new C-C bond between

the two isocyano carbon atoms, whereas the N-C cyclization and the 1,5-H shift steps proceeds simultaneously, one at each of the isocyanide molecules, thus leading to the betaine 4 through potential transition structures described as **TS1-4**. A final cyclization step at the electronegative moiety by using the electropositive one as the leaving group (either in a concert-ed manner through **TS4-2** – as represented - or stepwise via the pertinent tetrahedral intermediate) would give the final benzo-fused heteroazacycle **2** and the recovering of one molecule of the original isocyanide **1**. In this way, one molecule of **1** would assist the conversion of a second one by temporarily activating the isocyanide carbon of this latter, incrementing its nucleophilicity and thus assisting its prototropic 1,5-H shift. Unfortunately, in our hands the transition structure of the proposed first step was not located, probably due to its multiple breaking and forming bonds, a serious difficulty for the prevalence of a concerted process.

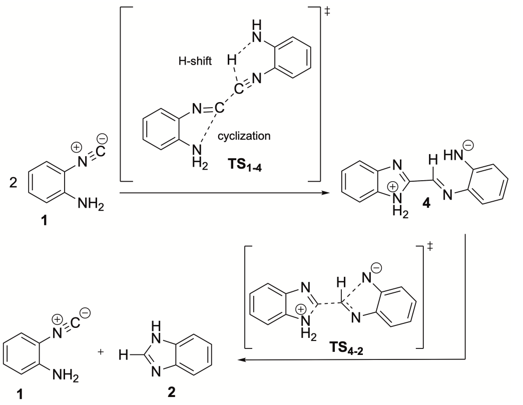

Scheme S1. Concerted bimolecular mechanistic proposal for the transformation of o-isocyanoaniline **1** into benzimidazole **2**.

Taking as computational model **1** (X = NH), and admitting its initial dimerization to 1,4-diazabutatriene **INT1** (Scheme S2) two routes emerge henceforth depending on how the carbon atoms of the NCCN fragment act either as nucleophilic (green pathway) or electrophilic (blue pathway). The green route starts by the transfer of one H atom of the amino group to the "purple" C atom, forming **INT2** and then cyclizing to **INT3**, in equilibrium with **INT7** and **INT4**. The latter one, colored in red, would experiment a new cyclization to **INT5**, further dissociating to benzimidazole **2** (X = NH) and **NHC**, the carbene 1,3-dihydro-2*H*-benzimidazol-2-ylidene.

Alternatively, from diazabutatriene **INT1**, the process might progress by the attack of the amino group at the "orange" C atom forming the five-membered ring of **INT6**, in prototropic equilibrium with **INT8**. Then, a second ring-closure by forming a new N-C bond would yield **INT9**, in equilibrium with **INT10**. This latter intermediate could dissociate into two molecules of carbene **NHC** transforming afterwards into benzimidazole **2** (X = NH)**.**

Note that green and blue routes might communicate via red **INT4**, coming from **INT8** via a prototropic step involving the amino group. In parallel, an additional brown route can be drawn if **INT6** converts into **INT11** by a 1,3-proton shift from the ammonium group to the central carbon atom of the anionic ketenimine fragment. A further cyclization of **INT11** would form **INT12** in equilibrium with **INT5**.

Under these lines we show a summary of all the mechanistic routes that we computed indicating the relative Gibss free energy of all compounds (kcal mol^{-1})**.**

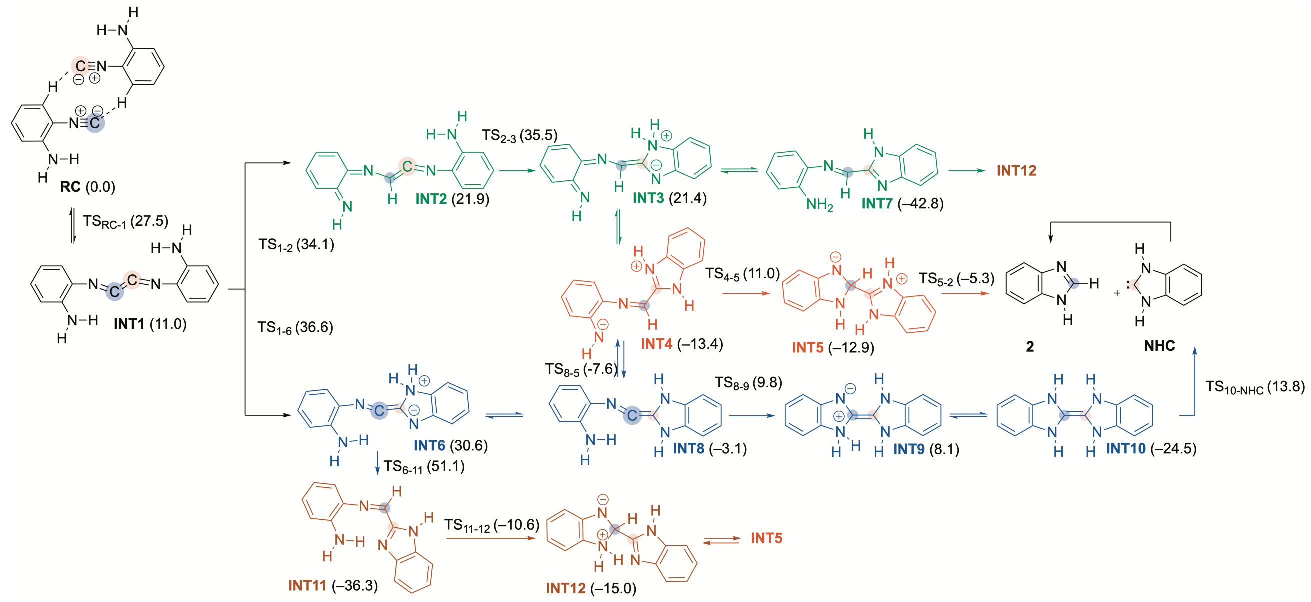

Scheme S2. Computed mechanisms for the transformation of o-isocyanoaniline **1** to benzimidazole **2** involving the 1,4-diazabutatriene **INT1**.

1.4. IRC calculations

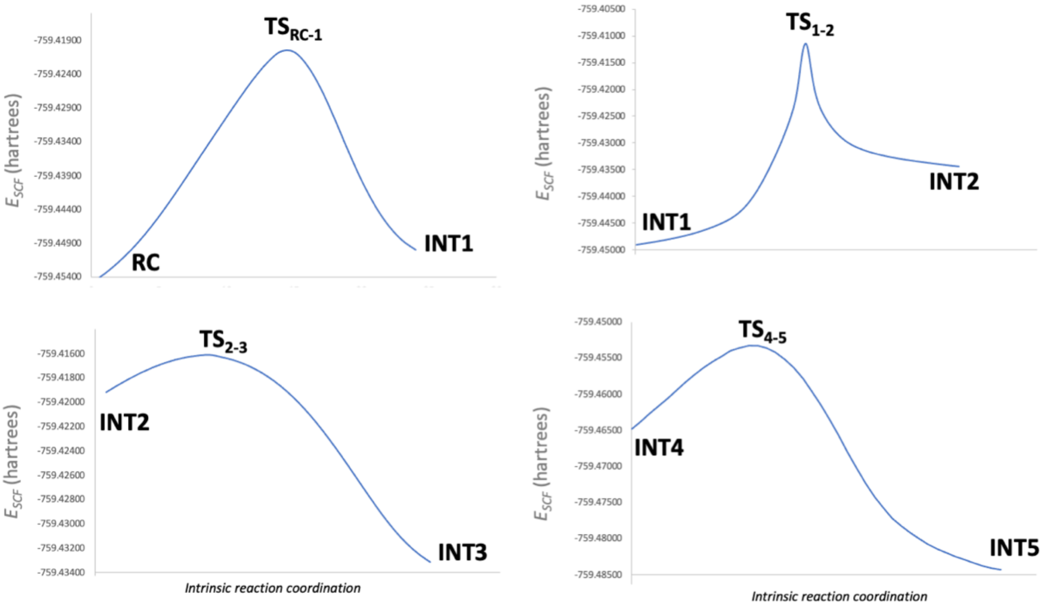

Figure S2. Plots of the IRC analysis for the transition structures **TS$_{RC-1}$, TS$_{1-2}$, TS$_{2-3}$, TS$_{4-5}$.**

1.5. Computational data

Table S2. Value of imaginary frequencies, electronic ($E_{SCF,298}$), thermochemical corrections (G_{corr}) (in Hartrees) of all stationary points found for the compounds shown in main text and in Scheme S2. Corrected Gibss free energies in solution at 298.15 K ($G_{298,sol}$) were calculated by the addition of the thermochemical corrections (G_{corr}) computed at M06-2x/aug-CC-pvDZ to the electronic energy computed at PCM($CHCl_3$)/M06-2x/aug-CC-pvTZ ($E_{SCF,298,sol}$).

Stationary Point	Imagin. Freq.	M06-2x/aug-CC-pvDZ		PCM($CHCl_3$)/M06-2x/aug-CC-pvTZ	
		$E_{SCF,298}$	G_{corr}	$E_{SCF,298,sol}$	$G_{298,sol}$
**1** (X=O)	-	-399.5918590	0.0721540	-399.6815449	-399.6093909
**1** (X=NH)	-	-379.7267342	0.0839822	-379.8124858	-379.7285036
**1** (X=NCO_2Me)	-	-607.5595137	0.1212357	-607.7011007	-607.5798650
**TS$_{conc}$** (X=O)	-1209.4	-399.5129639	0.0682539	-399.6020153	-399.5337614
**TS$_{conc}$** (X=NH)	-1241.6	-379.6322532	0.0797052	-379.7143218	-379.6346166
**TS$_{conc}$** (X=NCO_2Me)	-1278.8	-607.4716623	0.1171213	-607.6107723	-607.4936510
**2** (X=O)	-	-399.6284833	0.0768713	-399.7167528	-399.6398815
**2** (X=NH)	-	-379.7742160	0.0893360	-379.8596098	-379.7702738
**2** (X=NCO_2Me)	-	-607.6059734	0.1263094	-607.7448367	-607.6185273
**RC**	-	-759.4598833	0.1844013	-759.6276898	-759.4432885
**INT1**	-	-759.4511776	0.1895116	-759.6153292	-759.4258176
**INT2**	-	-759.4358453	0.1908483	-759.599198	-759.4083497
**INT3**	-	-759.4387457	0.1975567	-759.6067573	-759.4092006
**INT4**	-	-759.4971987	0.1981597	-759.6628276	-759.4646679
**INT5**	-	-759.4936831	0.2003111	-759.6641532	-759.4638421
**INT6**	-	-759.4165627	0.1940750	-759.5885316	-759.3944566
**INT7**	-	-759.5461427	0.1973557	-759.7088616	-759.5115059
**INT8**	-	-759.4765976	0.1940056	-759.6422729	-759.4482673
**INT9**	-	-759.4603574	0.1996974	-759.6300707	-759.4303733
**INT10**	-	-759.5169589	0.1977379	-759.6800792	-759.4823413
**INT11**	-	-759.5321993	0.1965953	-759.6977572	-759.5011619
**INT12**	-	-759.5021926	0.2016326	-759.6687568	-759.4671242
**NHC**	-	-379.7372987	0.0896547	-379.8221616	-379.7325069
**TS$_{RC-1}$**	-542.2	-759.4200004	0.1850994	-759.5845156	-759.3994162
**TS$_{1-2}$**	-1588.7	-759.4114366	0.1851896	-759.5740908	-759.3889012
**TS$_{2-3}$**	-271.6	-759.4161067	0.1926157	-759.5793438	-759.3867281
**TS$_{1-6}$**	-406.61	-759.408474	0.1920662	-759.5770805	-759.3850143
**TS$_{8-9}$**	-374.1	-759.4448153	0.1969663	-759.6107566	-759.4137903
**TS$_{8-4}$**	-1332.4	-759.4698774	0.1938954	-759.6345112	-759.4406158
**TS$_{4-5}$**	-414.4	-759.4531507	0.1971027	-759.6229054	-759.4258027
**TS$_{5-2}$**	-338.0	-759.4828630	0.1979590	-759.6496561	-759.4516971
**TS$_{10-NHC}$**	-416.3	-759.4521433	0.195907	-759.61717040	-759.4212634
**TS$_{6-11}$**	-1452.1	-759.3854150	0.1897350	-759.5516316	-759.3618966
**TS$_{11-12}$**	-381.3	-759.4962325	0.1997025	-759.6598405	-759.4601380
**TSa**	-1577.4	-379.6628141	0.0834791	-379.7451782	-379.6616991

**TSb**	-1380.1	-759.4818935	0.1926765	-759.642628	-759.4499515
**RC** (X=O)	-	-799.190729	0.161002	-799.3662843	-799.2052823
**TS$_{RC-1}$** (X=O)	-540.6	-799.153882	0.163115	-799.3264585	-799.1633435
**INT1** (X=O)	-	-799.191058	0.1681346	-799.3638935	-799.1957589
**RC** (X=NCO$_2$Me)	-	-1215.13613	0.2594485	-1215.413491	-1215.1540421
**TS$_{RC-1}$** (X=NCO$_2$Me)	-544.7	-1215.09861	0.2626348	-1215.372863	-1215.1102277
**INT1** (X=NCO$_2$Me)	-	-1215.13384	0.2672363	-1215.408492	-1215.1412559

1.6. Cartesian Coordinates

The lowest energy conformer is shown. For all calculated structures a charge of 0 and multiplicity of 1 were applied. Cartesian coordinates are given in Ångströms (Å).

1 (X=O)

```
C	-1.344444	1.162033	0.000114
C	-2.250444	0.107834	0.000051
C	-1.809709	-1.219228	-0.000058
C	-0.447861	-1.489811	-0.000105
C	0.463666	-0.432011	-0.000042
C	0.026790	0.901913	0.000067
H	-1.673374	2.198899	0.000199
H	-3.317526	0.323991	0.000087
H	-2.525694	-2.037783	-0.000105
H	-0.066852	-2.508799	-0.000186
N	1.834323	-0.663177	-0.000084
C	3.004566	-0.782054	-0.000118
H	1.797710	1.621276	0.000091
O	0.886261	1.944075	0.000130
```

1 (X=NH)

```
C	-1.334041	1.162041	0.001340
C	-2.254072	0.121838	0.006424
C	-1.833401	-1.211414	0.003186
C	-0.473097	-1.491505	-0.001285
C	0.451615	-0.446771	-0.005414
C	0.045428	0.902066	-0.005009
H	-1.674836	2.196817	-0.002941
H	-3.317945	0.354366	0.011514
H	-2.558357	-2.021781	0.005957
H	-0.102115	-2.514319	-0.000436
N	1.815513	-0.713493	-0.000306
C	2.981702	-0.861367	0.007913
N	0.977381	1.916634	-0.068249
H	0.671553	2.829828	0.232607
H	1.926643	1.683780	0.190254
```

1 (X=NCO$_2$Me)

```
C	-0.403578	-1.494257	-0.000053
C	-1.572514	-2.250456	-0.000039
C	-2.830459	-1.649590	-0.000006
C	-2.913942	-0.263326	0.000017
C	-1.747853	0.499337	0.000006
C	-0.466362	-0.094185	-0.000030
H	0.558216	-1.989834	-0.000080
H	-1.490983	-3.336120	-0.000058
H	-3.735847	-2.251964	0.000003
H	-3.871350	0.252725	0.000045
N	-1.841346	1.886423	0.000027
C	-1.886648	3.061728	0.000045
N	0.629033	0.771224	-0.000041
H	0.418162	1.761980	-0.000080
C	1.989335	0.553529	0.000010
O	2.784597	1.464600	-0.000057
O	2.327661	-0.744083	0.000067
C	3.742419	-0.975612	0.000054
H	4.194712	-0.533395	0.892997
H	3.858081	-2.060942	-0.000101
H	4.194749	-0.533125	-0.892734
```

TS$_{conc}$ (X=O)

```
C	0.971427	1.381550	-0.000138
C	2.125361	0.614062	-0.000011
C	2.093894	-0.790685	0.000128
C	0.869552	-1.442612	0.000089
C	-0.284395	-0.668250	-0.000190
C	-0.305653	0.764498	-0.000172
H	1.013451	2.468129	-0.000140
H	3.089346	1.122205	0.000008
H	3.018603	-1.362372	0.000323
H	0.791769	-2.527755	0.000223
N	-1.608995	-1.165710	0.000065
C	-2.547263	-0.445556	0.000206
H	-2.809095	0.658016	0.000110
O	-1.422332	1.415464	-0.000057
```

TS$_{conc}$ (X=NH)

```
C	-0.983762	1.384702	0.021345
C	-2.135733	0.624561	0.021308
C	-2.105934	-0.786440	0.000867
C	-0.886185	-1.437323	-0.018832
C	0.280459	-0.671101	-0.022909
C	0.297287	0.759483	-0.018453
H	-1.040859	2.472074	0.027344
H	-3.099262	1.133472	0.037075
H	-3.032947	-1.354437	0.011027
H	-0.811074	-2.522901	-0.023047
N	1.574348	-1.190231	0.023025
C	2.558937	-0.509769	0.002526
N	1.491812	1.359900	-0.071684
H	1.482783	2.367800	0.058332
H	2.887825	0.531631	0.314772
```

TS$_{conc}$ (X=NCO$_2$Me)

```
C	-0.659882	-1.443822	-0.000053
C	-1.918151	-2.032889	-0.000112
C	-3.098645	-1.279937	-0.000138
C	-3.012958	0.105566	-0.000102
C	-1.754744	0.691887	-0.000042
```

C	-0.527662	-0.035920	-0.000015
H	0.232094	-2.058768	-0.000036
H	-1.979831	-3.120553	-0.000140
H	-4.069057	-1.770458	-0.000184
H	-3.895936	0.740806	-0.000119
N	-1.555557	2.092043	0.000001
C	-0.483502	2.603383	0.000054
N	0.604537	0.720914	0.000047
H	0.634916	2.343836	0.000092
C	1.923923	0.377755	0.000078
O	2.808468	1.214271	0.000137
O	2.172289	-0.949937	0.000036
C	3.563103	-1.286440	0.000070
H	3.599325	-2.377919	0.000027
H	4.050374	-0.879866	-0.891913
H	4.050314	-0.879938	0.892118

2 (X=O)

C	-0.897781	-1.432463	-0.000105
C	-2.088963	-0.710210	-0.000048
C	-2.103082	0.697476	0.000046
C	-0.926598	1.439738	0.000088
C	0.274512	0.727210	0.000049
C	0.261609	-0.669489	-0.000046
H	-0.876962	-2.519496	-0.000177
H	-3.034605	-1.249535	-0.000080
H	-3.061629	1.213418	0.000081
H	-0.930456	2.527478	0.000155
N	1.606312	1.158953	0.000064
C	2.281168	0.064511	0.000033
H	3.359271	-0.048390	0.000022
O	1.561876	-1.092099	-0.000068

2 (X=NH)

C	-0.923055	1.434186	-0.000143
C	-2.110575	0.715183	-0.000020
C	-2.120749	-0.695831	0.000100
C	-0.942824	-1.428331	0.000011
C	0.267705	-0.722746	-0.000001
C	0.258941	0.687977	0.000059
H	-0.920090	2.522772	-0.000255
H	-3.057436	1.252690	-0.000036
H	-3.077283	-1.215718	0.000214
H	-0.943405	-2.516510	0.000028
N	1.572298	-1.197915	-0.000132
C	2.314650	-0.125633	-0.000007
N	1.593556	1.042191	0.000025
H	1.972896	1.976037	0.000666
H	3.399774	-0.118036	0.000149

2 (X=NCO$_2$Me)

C	-0.838258	-1.481358	-0.000326
C	-2.169397	-1.886985	-0.000142
C	-3.228739	-0.962086	0.000123
C	-2.987670	0.405964	0.000190
C	-1.657822	0.831796	0.000062
C	-0.606479	-0.105379	-0.000171
H	-0.027012	-2.201156	-0.000548
H	-2.394937	-2.952114	-0.000202
H	-4.253716	-1.328856	0.000253
H	-3.795146	1.134973	0.000358
N	-1.159041	2.136465	0.000132
C	0.126914	2.006911	-0.000091
H	0.860233	2.805572	-0.000175
N	0.552649	0.680139	-0.000313
C	1.902348	0.330225	-0.000103
O	2.794253	1.140036	0.000225
O	2.056434	-0.994685	-0.000113
C	3.425278	-1.425572	0.000346
H	3.385090	-2.515767	0.000711
H	3.934044	-1.051700	-0.893484
H	3.933646	-1.051074	0.894135

RC

C	-5.288968	0.094381	0.000909
C	-5.235989	-1.293843	-0.005119
C	-4.011298	-1.967800	-0.013794
C	-2.830799	-1.235432	-0.012554
C	-2.887875	0.158666	-0.005779
C	-4.110984	0.856248	0.000589
H	-6.249500	0.608509	0.000248
H	-6.166499	-1.859835	-0.004621
H	-3.977789	-3.054573	-0.019623
H	-1.853062	-1.714281	-0.014740
N	-1.715686	0.907034	0.004681
C	-0.755842	1.582184	0.016998
N	-4.136473	2.236717	-0.052146
H	-4.984646	2.679209	0.269200
H	-3.288987	2.715529	0.221482
C	0.755925	-1.582382	0.016284
N	1.715761	-0.907211	0.004442
C	2.887880	-0.158732	-0.005990
C	2.830666	1.235356	-0.012685
C	4.111059	-0.856197	0.000357
C	4.011097	1.967838	-0.013856
H	1.852879	1.714108	-0.014853
C	5.288968	-0.094219	0.000744
N	4.136681	-2.236662	-0.052452
C	5.235853	1.294002	-0.005201
H	3.977479	3.054608	-0.019618
H	6.249550	-0.608253	0.000073
H	4.984892	-2.679090	0.268879
H	3.289238	-2.715569	0.221143
H	6.166309	1.860083	-0.004651

INT1

C	4.727666	1.169830	0.005914
C	5.671974	0.158513	-0.025656
C	5.280555	-1.188014	-0.043900
C	3.930379	-1.486934	-0.031761
C	2.947750	-0.480949	-0.001957
C	3.346974	0.883819	0.019218
H	5.042862	2.213186	0.022551
H	6.729623	0.418937	-0.035639
H	6.023632	-1.981609	-0.067708
H	3.577178	-2.516564	-0.045637
N	1.628857	-0.934577	0.003553
C	0.608390	-0.204650	0.009546
N	2.447342	1.910768	0.082334
H	2.786679	2.854218	-0.010462
H	1.458406	1.753525	-0.031939
C	-0.608403	0.204777	0.009455
N	-1.628899	0.934655	0.003376
C	-2.947771	0.480970	-0.002453
C	-3.930437	1.486918	-0.032272
C	-3.346940	-0.883820	0.018424
C	-5.280598	1.187943	-0.044707
H	-3.577277	2.516565	-0.045914
C	-4.727620	-1.169886	0.004820
N	-2.447283	-1.910743	0.081506
C	-5.671963	-0.158604	-0.026756
H	-6.023704	1.981511	-0.068521
H	-5.042774	-2.213258	0.021226
H	-2.786563	-2.854201	-0.011411

H	-1.458319	-1.753448	-0.032440
H	-6.729600	-0.419070	-0.036975

INT2

C	-4.986436	-0.408981	0.594032
C	-5.506947	0.674148	-0.019939
C	-4.678893	1.557477	-0.819541
C	-3.360244	1.300376	-0.959854
C	-2.714743	0.153534	-0.333086
C	-3.563361	-0.759844	0.494459
H	-5.614330	-1.069247	1.193481
H	-6.569233	0.895977	0.081617
H	-5.131849	2.421211	-1.302439
H	-2.710675	1.939762	-1.555288
N	-1.435744	0.047508	-0.569936
C	-0.642358	-0.953025	-0.079701
N	-3.051713	-1.791066	1.080470
H	-3.782153	-2.287969	1.596009
H	-0.989972	-1.756328	0.564281
C	0.663304	-0.898737	-0.426020
N	1.804240	-0.922472	-0.802896
C	3.029256	-0.373428	-0.392806
C	4.171403	-0.748223	-1.105179
C	3.112622	0.535206	0.685333
C	5.418305	-0.247991	-0.760283
H	4.047010	-1.444330	-1.932375
C	4.384416	1.035274	1.016515
N	2.005752	0.879341	1.430359
C	5.513358	0.648689	0.309169
H	6.304346	-0.546594	-1.315257
H	4.471309	1.735334	1.847232
H	2.108818	1.685298	2.029189
H	1.087886	0.792123	1.015064
H	6.483012	1.054731	0.593904

INT3

C	4.882496	-0.567116	-0.000003
C	5.324075	0.713360	-0.000000
C	4.400812	1.821043	0.000002
C	3.065488	1.572327	0.000000
C	2.521302	0.236318	-0.000000
C	3.458327	-0.909991	0.000002
H	5.588206	-1.398915	-0.000006
H	6.395281	0.915706	-0.000000
H	4.779541	2.841047	0.000001
H	2.342597	2.388031	-0.000001
N	1.197171	0.159444	-0.000000
C	0.475471	-0.968640	0.000001
N	3.019128	-2.132929	-0.000006
H	3.814261	-2.775494	-0.000011
H	0.884061	-1.973667	0.000002
C	-0.910188	-0.773059	0.000001
N	-1.954225	-1.548129	0.000001
C	-3.090527	-0.752168	0.000001
C	-4.431514	-1.141573	0.000001
C	-2.794658	0.619927	0.000000
C	-5.406068	-0.141883	0.000001
H	-4.694339	-2.196597	0.000002
C	-3.738340	1.624087	0.000000
N	-1.340008	0.712768	-0.000000
C	-5.079545	1.219018	0.000001
H	-6.456428	-0.429782	0.000002
H	-3.465864	2.678244	-0.000001
H	-0.918314	1.167743	-0.815144
H	-0.918314	1.167744	0.815144
H	-5.867938	1.967959	0.000001

INT4

C	-4.455369	-1.159026	0.000054
C	-5.455776	-0.183454	0.000078
C	-5.143480	1.180621	0.000069
C	-3.819345	1.626049	0.000038
C	-2.824681	0.656556	0.000022
C	-3.140101	-0.714995	0.000030
H	-4.698837	-2.219326	0.000055
H	-6.498906	-0.492806	0.000101
H	-5.949097	1.912059	0.000086
H	-3.577928	2.686530	0.000028
N	-1.440993	0.738604	-0.000022
C	-0.886988	-0.504581	-0.000022
N	-1.925235	-1.392150	-0.000018
H	-1.794357	-2.392197	0.000074
H	-0.844232	1.555589	0.000003
C	0.482839	-0.786528	-0.000031
N	1.266122	0.296569	-0.000023
C	2.589884	0.287200	-0.000024
C	3.217201	1.587528	-0.000024
C	3.452008	-0.916705	-0.000029
C	4.565127	1.751399	-0.000026
H	2.543452	2.443768	-0.000022
C	4.894150	-0.663925	-0.000031
N	2.940577	-2.113329	-0.000024
C	5.415153	0.586782	-0.000030
H	5.008196	2.745116	-0.000025
H	5.547262	-1.537800	-0.000034
H	3.699600	-2.798169	-0.000026
H	0.840482	-1.812041	-0.000042
H	6.497328	0.719889	-0.000032

INT5

C	-3.471353	-0.664004	1.224756
C	-4.409754	0.351710	1.089041
C	-4.241746	1.394327	0.158451
C	-3.128842	1.460730	-0.670153
C	-2.184883	0.443706	-0.535307
C	-2.354803	-0.598631	0.392944
H	-3.601767	-1.468198	1.945198
H	-5.297317	0.341564	1.718439
H	-5.003064	2.168571	0.088383
H	-2.996317	2.267305	-1.387121
N	-0.977020	0.193342	-1.173133
C	-0.429861	-0.908856	-0.681896
N	-1.235793	-1.417491	0.254437
H	-0.939758	-2.192185	0.836621
H	-0.377467	0.726940	-1.816670
C	0.940569	-1.387454	-1.169384
N	1.644903	-1.834847	0.052409
C	2.366137	-0.623933	0.389106
C	3.059833	-0.333804	1.544825
C	2.308718	0.252364	-0.733415
C	3.744024	0.891659	1.620815
H	3.078982	-1.040520	2.374785
C	3.003777	1.473446	-0.638434
N	1.579172	-0.251460	-1.775280
C	3.705532	1.768930	0.533155
H	4.295754	1.155170	2.520734
H	3.001375	2.166930	-1.478153
H	0.766642	-2.227744	-1.857800
H	4.238901	2.717742	0.600811
H	2.311104	-2.563522	-0.201291

INT6

C	3.899343	0.393158	1.452128

C	5.110043	0.627772	0.791806
C	5.188216	0.407909	-0.594526
C	4.112134	-0.035016	-1.354054
C	2.873309	-0.278654	-0.715633
C	2.844019	-0.042111	0.679882
H	3.803051	0.550726	2.526427
H	5.977394	0.974867	1.346882
H	6.137647	0.592536	-1.097306
H	4.205494	-0.199657	-2.424916
N	1.709354	-0.710284	-1.247161
C	0.809321	-0.779030	-0.225526
N	1.475064	-0.373292	1.116684
H	1.446619	-1.153450	1.777969
H	0.981690	0.420193	1.531565
C	-0.498480	-0.983288	-0.151055
N	-1.660104	-1.386083	0.020071
C	-2.811643	-0.562861	-0.002542
C	-4.024134	-1.231535	0.205997
C	-2.807197	0.840022	-0.189058
C	-5.231639	-0.550122	0.247960
H	-3.976831	-2.311059	0.338482
C	-4.037028	1.516822	-0.137167
N	-1.630020	1.563832	-0.351677
C	-5.227781	0.837461	0.078541
H	-6.163315	-1.087192	0.410880
H	-4.043605	2.598516	-0.274920
H	-1.778344	2.505452	-0.691262
H	-0.878133	1.091223	-0.838948
H	-6.162623	1.395475	0.109218

INT7

C	-3.987810	-1.394106	0.685733
C	-5.252173	-0.876016	0.445817
C	-5.436722	0.369495	-0.195087
C	-4.359494	1.133009	-0.615056
C	-3.070428	0.630109	-0.383576
C	-2.903705	-0.619374	0.259893
H	-3.851728	-2.354233	1.179414
H	-6.126725	-1.443806	0.759373
H	-6.450035	0.731410	-0.360136
H	-4.493026	2.092734	-1.109955
N	-1.839963	1.177048	-0.695050
C	-0.967511	0.297522	-0.259981
N	-1.542308	-0.800096	0.323614
H	-1.013610	-1.571793	0.705715
H	1.807215	1.791327	1.279939
C	0.483089	0.435820	-0.364497
N	1.231656	-0.485592	0.106717
C	2.638219	-0.367604	0.009217
C	3.343635	-1.503961	-0.398788
C	3.346123	0.800661	0.370145
C	4.727515	-1.490051	-0.528362
H	2.768350	-2.399988	-0.626430
C	4.742848	0.794438	0.253150
N	2.691528	1.956856	0.813528
C	5.426295	-0.327241	-0.201412
H	5.255556	-2.378089	-0.869079
H	5.293932	1.694939	0.525721
H	3.286047	2.560877	1.366185
H	0.846955	1.338835	-0.869041
H	6.511402	-0.295926	-0.286536

INT8

C	4.226071	-0.341128	-1.384778
C	5.409019	-0.001777	-0.714173
C	5.398005	0.373965	0.629198
C	4.203607	0.426309	1.360404
C	3.034736	0.090318	0.695578
C	3.045715	-0.288144	-0.660207
H	4.236651	-0.637195	-2.431669
H	6.352897	-0.033863	-1.254712
H	6.333381	0.631225	1.122166
H	4.196587	0.715363	2.409261
N	1.711610	0.018775	1.115214
C	0.883182	-0.337747	0.067130
N	1.728186	-0.568504	-1.002990
H	1.393348	-0.857707	-1.907950
H	1.358854	0.255794	2.028654
C	-0.482894	-0.184502	0.018343
N	-1.496654	-0.913033	0.244616
C	-2.822029	-0.441880	0.118017
C	-3.812347	-1.413938	0.316517
C	-3.213265	0.890026	-0.179285
C	-5.165070	-1.120432	0.219605
H	-3.468518	-2.420767	0.549030
C	-4.588614	1.174719	-0.275912
N	-2.295314	1.905903	-0.321537
C	-5.546567	0.190688	-0.082925
H	-5.912450	-1.895319	0.375573
H	-4.892893	2.196870	-0.503912
H	-2.618782	2.756380	-0.756983
H	-1.329503	1.639388	-0.475037
H	-6.601672	0.448978	-0.166613

INT9

C	-3.957389	-1.393792	-0.292160
C	-5.083630	-0.613108	-0.586769
C	-5.041654	0.777713	-0.517030
C	-3.854764	1.439175	-0.163727
C	-2.744283	0.668137	0.117794
C	-2.789192	-0.734331	0.064097
H	-4.000712	-2.480336	-0.333553
H	-6.010119	-1.107843	-0.873763
H	-5.931989	1.358806	-0.748523
H	-3.806681	2.526135	-0.124727
N	-1.424378	1.086370	0.495170
C	-0.667036	-0.132582	0.526965
N	-1.549095	-1.234438	0.474102
H	-1.176539	-2.095038	0.091424
H	-1.444359	1.563898	1.395745
C	0.671925	-0.207428	0.464097
N	1.527644	-1.263940	0.404534
C	2.736866	-0.752521	0.084273
C	3.960722	-1.416677	-0.165938
C	2.773520	0.661692	-0.003901
C	5.089997	-0.663450	-0.461723
H	4.001701	-2.502637	-0.122713
C	3.886091	1.427496	-0.279421
N	1.414425	1.120229	0.341687
C	5.080375	0.741672	-0.523253
H	6.027340	-1.185460	-0.654724
H	3.843869	2.516197	-0.315960
H	0.947093	1.701076	-0.363196
H	1.412841	1.644986	1.220855
H	5.988097	1.290691	-0.759135

INT10

C	-4.010563	-1.418008	-0.013420
C	-5.213371	-0.696498	0.017706
C	-5.213418	0.696363	0.017749
C	-4.010648	1.417942	-0.013322
C	-2.825781	0.701525	-0.032453
C	-2.825731	-0.701512	-0.032503

H	-4.008631	-2.506604	-0.016550
H	-6.158503	-1.235952	0.038166
H	-6.158582	1.235758	0.038249
H	-4.008781	2.506539	-0.016375
N	-1.492199	1.160208	-0.080751
C	-0.667033	0.000084	0.000511
N	-1.492138	-1.160054	-0.080887
H	-1.249048	-1.909320	0.560210
H	-1.249297	1.909222	0.560718
C	0.667129	0.000070	0.014464
N	1.492791	-1.159216	0.094212
C	2.826052	-0.701524	0.034488
C	4.010610	-1.418068	0.006317
C	2.826079	0.701573	0.034492
C	5.213168	-0.696487	-0.034139
H	4.008729	-2.506662	0.009419
C	4.010713	1.418029	0.006253
N	1.492908	1.159200	0.094315
C	5.213198	0.696341	-0.034180
H	6.158112	-1.235929	-0.062074
H	4.008948	2.506623	0.009284
H	1.243408	1.912897	-0.538934
H	6.158175	1.235727	-0.062159
H	1.243516	-1.912250	-0.539959

INT11

C	-4.150545	0.541756	-0.291845
C	-4.680184	-0.738594	-0.345427
C	-3.862967	-1.886709	-0.239219
C	-2.491148	-1.786190	-0.076362
C	-1.933047	-0.500276	-0.020170
C	-2.764401	0.636376	-0.126023
H	-4.784461	1.422344	-0.374659
H	-5.754049	-0.863993	-0.473405
H	-4.328268	-2.869501	-0.289176
H	-1.854426	-2.664856	0.001019
N	-0.614232	-0.118150	0.132482
C	-0.631880	1.193849	0.119480
N	-1.904377	1.705060	-0.024854
H	-2.143838	2.684005	-0.078548
H	0.712399	-0.041369	1.918619
C	0.502134	2.129447	0.213587
N	1.747078	1.922448	0.082668
C	2.374014	0.673645	-0.113200
C	3.152800	0.541764	-1.268433
C	2.402061	-0.340181	0.868393
C	3.922899	-0.593067	-1.494927
H	3.145564	1.361880	-1.984519
C	3.215849	-1.457545	0.640704
N	1.704917	-0.186196	2.076285
C	3.964322	-1.592702	-0.523175
H	4.504179	-0.683959	-2.410387
H	3.244318	-2.241066	1.398617
H	1.827676	-0.990509	2.679722
H	0.198976	3.174970	0.356616
H	4.578775	-2.479520	-0.670255

INT12

C	-3.445154	-1.123455	0.889247
C	-4.460198	-0.215507	1.155019
C	-4.400034	1.114477	0.689264
C	-3.324521	1.576278	-0.057465
C	-2.306173	0.657441	-0.322555
C	-2.350849	-0.677946	0.137929
H	-3.488464	-2.150211	1.246814
H	-5.324952	-0.532104	1.735639
H	-5.217526	1.795021	0.921106
H	-3.280311	2.602670	-0.415782
N	-1.113991	0.751877	-1.013807
C	-0.521230	-0.464906	-0.940233
N	-1.207730	-1.356356	-0.267963
H	0.801311	-2.124423	-0.013358
H	-0.645735	1.554608	-1.416881
C	0.823157	-0.708729	-1.593066
N	1.533498	-1.661263	-0.568637
C	2.361917	-0.692175	0.169683
C	3.103984	-0.916869	1.311486
C	2.366004	0.498604	-0.602123
C	3.901221	0.134425	1.770968
H	3.065299	-1.868334	1.841873
C	3.193825	1.540471	-0.114189
N	1.611796	0.457017	-1.720560
C	3.930675	1.338504	1.045409
H	4.488842	0.021129	2.678140
H	3.245102	2.482233	-0.656379
H	0.690498	-1.271555	-2.526588
H	4.559136	2.151102	1.410623
H	2.106053	-2.362747	-1.044693

NHC

C	0.923732	1.427855	-0.000057
C	2.112287	0.702486	0.000221
C	2.112280	-0.702541	0.000217
C	0.923698	-1.427910	-0.000048
C	-0.265347	-0.700519	-0.000631
C	-0.265380	0.700504	-0.000707
H	0.926611	2.516321	0.000064
H	3.061896	1.234491	0.000572
H	3.061893	-1.234524	0.000522
H	0.926464	-2.516368	0.000074
N	-1.608691	-1.061592	0.000070
C	-2.468266	-0.000011	0.000615
N	-1.608605	1.061665	0.000215
H	-1.946579	2.011540	-0.000630
H	-1.947236	-2.011160	-0.000261

TS_{RC-1}

C	-5.213468	-0.180234	0.324081
C	-5.267777	1.152648	-0.064158
C	-4.114889	1.830705	-0.473626
C	-2.901283	1.154284	-0.492668
C	-2.845443	-0.184685	-0.105058
C	-3.996940	-0.879699	0.308590
H	-6.116919	-0.695972	0.648084
H	-6.225360	1.671548	-0.049249
H	-4.166511	2.872982	-0.780275
H	-1.980310	1.643046	-0.807136
N	-1.634156	-0.890011	-0.142059
C	-0.442297	-0.743876	-0.224838
N	-3.904085	-2.189145	0.743568
H	-4.749604	-2.737560	0.686067
H	-3.067388	-2.681350	0.458792
C	0.442292	0.743854	-0.224874
N	1.634152	0.889996	-0.142107
C	2.845441	0.184676	-0.105079
C	2.901283	-1.154316	-0.492611
C	3.996940	0.879719	0.308516
C	4.114891	-1.830730	-0.473543
H	1.980307	-1.643099	-0.807041
C	5.213471	0.180260	0.324034
N	3.904085	2.189189	0.743419
C	5.267781	-1.152645	-0.064127
H	4.166514	-2.873026	-0.780131
H	6.116924	0.696019	0.647997

H	4.749601	2.737606	0.685875
H	3.067382	2.681375	0.458625
H	6.225366	-1.671540	-0.049199

TS$_{1-2}$

C	4.939830	-0.789610	0.461629
C	5.624340	0.346677	0.130961
C	4.961232	1.495863	-0.395715
C	3.604812	1.462043	-0.563616
C	2.838186	0.301802	-0.242170
C	3.517260	-0.879938	0.272109
H	5.461103	-1.658522	0.863410
H	6.704629	0.376888	0.274073
H	5.534366	2.384719	-0.649484
H	3.053527	2.318968	-0.948566
N	1.501799	0.387757	-0.400954
C	0.769621	-0.684930	-0.087991
N	2.802154	-1.960928	0.541360
H	3.317121	-2.728864	0.963619
H	1.536317	-1.659043	0.353145
C	-0.579486	-0.667640	-0.242431
N	-1.741565	-0.872417	-0.380447
C	-3.047111	-0.448310	-0.184794
C	-4.107652	-1.311061	-0.471877
C	-3.272760	0.867472	0.280662
C	-5.417392	-0.892778	-0.286091
H	-3.875805	-2.309316	-0.837327
C	-4.607148	1.270037	0.452776
N	-2.224070	1.696931	0.614363
C	-5.657481	0.404861	0.177302
H	-6.243341	-1.565641	-0.503474
H	-4.808024	2.278103	0.814301
H	-2.448289	2.674624	0.725662
H	-1.317007	1.533480	0.195658
H	-6.680326	0.748277	0.324206

TS$_{2-3}$

C	-4.912011	-0.577446	-0.318965
C	-5.380175	0.671814	-0.102312
C	-4.488650	1.757311	0.245507
C	-3.159800	1.522498	0.354660
C	-2.575514	0.211846	0.136728
C	-3.487291	-0.912234	-0.215405
H	-5.590473	-1.390781	-0.579504
H	-6.448268	0.871698	-0.188548
H	-4.895574	2.751694	0.418024
H	-2.461711	2.315762	0.618397
N	-1.270818	0.154181	0.279670
C	-0.535526	-0.962045	0.086768
N	-3.027145	-2.106456	-0.415129
H	-3.799982	-2.734122	-0.648382
H	-0.941564	-1.909006	-0.266446
C	0.811968	-0.913042	0.310557
N	1.929731	-1.369950	0.539863
C	3.068760	-0.652249	0.169653
C	4.356077	-1.183670	0.201409
C	2.858636	0.711052	-0.134329
C	5.438086	-0.346199	-0.062149
H	4.494231	-2.235503	0.441001
C	3.950927	1.541677	-0.357682
N	1.491125	1.100642	-0.118904
C	5.242084	1.010031	-0.329396
H	6.447610	-0.753520	-0.045924
H	3.792077	2.597088	-0.575382
H	1.269685	1.897683	0.468901
H	1.052274	1.207808	-1.027188
H	6.096009	1.656214	-0.523707

TS$_{1-6}$

C	3.951756	0.873053	1.230130
C	5.120824	1.004112	0.475996
C	5.204360	0.397715	-0.783119
C	4.155916	-0.352995	-1.305704
C	2.973367	-0.492165	-0.563130
C	2.887854	0.172036	0.686966
H	3.869221	1.338028	2.212278
H	5.956040	1.582672	0.864152
H	6.117936	0.508537	-1.366344
H	4.237481	-0.844773	-2.272370
N	1.924399	-1.327803	-0.845677
C	0.886805	-1.055429	-0.115526
N	1.623038	-0.100858	1.327958
H	1.712544	-0.595495	2.216281
H	1.018630	0.706466	1.439109
C	-0.417770	-0.943021	-0.068409
N	-1.637229	-1.176144	0.077999
C	-2.609818	-0.157485	-0.080526
C	-2.311347	1.168606	-0.412615
C	-3.948795	-0.553473	0.128033
C	-3.315755	2.119724	-0.518738
H	-1.269236	1.434059	-0.592910
C	-4.958017	0.420380	0.023365
N	-4.252104	-1.874796	0.375927
C	-4.643342	1.734460	-0.290672
H	-3.076671	3.148590	-0.779203
H	-5.995315	0.126463	0.182062
H	-5.121422	-2.067744	0.848894
H	-3.469227	-2.462852	0.626750
H	-5.442934	2.470159	-0.368456

TS$_{8-9}$

C	-4.041337	-1.329535	-0.470597
C	-5.163524	-0.493209	-0.555068
C	-5.065604	0.873286	-0.303565
C	-3.834811	1.450763	0.045034
C	-2.732510	0.621172	0.139622
C	-2.829175	-0.754934	-0.116555
H	-4.119364	-2.396159	-0.672583
H	-6.126130	-0.922386	-0.827707
H	-5.950061	1.502591	-0.380618
H	-3.751326	2.518403	0.241606
N	-1.390895	0.944402	0.484436
C	-0.673821	-0.300646	0.445745
N	-1.562160	-1.332636	0.019147
H	-1.476013	-2.242467	0.453279
H	-1.321213	1.433719	1.371773
C	0.644912	-0.427536	0.588841
N	1.647991	-1.217984	0.757328
C	2.784314	-0.690161	0.184350
C	3.987017	-1.372690	-0.051291
C	2.750250	0.710776	-0.030253
C	5.108538	-0.659168	-0.458481
H	4.024689	-2.449440	0.099186
C	3.885867	1.426020	-0.384129
N	1.461669	1.270319	0.285359
C	5.074065	0.732568	-0.616778
H	6.038439	-1.193920	-0.648944
H	3.842414	2.508129	-0.507505
H	0.898810	1.582135	-0.502835
H	1.511985	2.018956	0.976858
H	5.966449	1.271495	-0.927646

TS$_{8-4}$

C	4.398212	1.189758	0.000370

C	5.419657	0.242491	0.000362
C	5.143533	-1.133941	0.000040
C	3.837007	-1.616770	-0.000318
C	2.814391	-0.671749	-0.000340
C	3.093010	0.707716	0.000067
H	4.612625	2.256190	0.000636
H	6.454843	0.577453	0.000621
H	5.970534	-1.841165	0.000065
H	3.624688	-2.683348	-0.000578
N	1.434784	-0.784443	-0.000574
C	0.856402	0.436581	-0.000203
N	1.858868	1.345066	-0.000147
H	1.675928	2.338801	0.000735
H	0.840089	-1.606869	-0.000925
C	-0.551425	0.724397	0.000287
N	-1.229875	-0.411426	0.000193
C	-2.600024	-0.361785	0.000265
C	-3.295570	-1.593752	0.000354
C	-3.345376	0.877144	-0.000032
C	-4.671025	-1.653595	0.000244
H	-2.690339	-2.500187	0.000502
C	-4.771802	0.778678	-0.000178
N	-2.676307	2.034351	-0.000290
C	-5.404430	-0.441995	-0.000004
H	-5.189186	-2.609969	0.000334
H	-5.351424	1.702311	-0.000399
H	-3.250360	2.870698	-0.000497
H	-1.450838	1.787811	-0.000168
H	-6.494208	-0.475629	-0.000071

TS$_{4-5}$

C	3.826956	-0.476205	0.577650
C	4.452586	0.755175	0.396742
C	3.786203	1.836923	-0.200017
C	2.468904	1.729512	-0.639087
C	1.844098	0.501214	-0.453270
C	2.509795	-0.578767	0.144247
H	4.344503	-1.313878	1.039743
H	5.481621	0.881164	0.727095
H	4.310701	2.782474	-0.321395
H	1.950787	2.567036	-1.100185
N	0.571358	0.041404	-0.775948
C	0.415713	-1.214987	-0.340192
N	1.598172	-1.633114	0.149547
H	1.721953	-2.533216	0.589115
H	-0.213515	0.568810	-1.146567
C	-0.817158	-2.009699	-0.473777
N	-1.874422	-1.563482	-1.128220
C	-2.329903	-0.380047	-0.576335
C	-3.199549	0.525375	-1.200281
C	-2.034002	-0.237416	0.826130
C	-3.835491	1.513424	-0.449947
H	-3.400303	0.411265	-2.264622
C	-2.755420	0.705533	1.566145
N	-1.039587	-1.071308	1.314170
C	-3.632074	1.588266	0.930763
H	-4.511770	2.213018	-0.939396
H	-2.578976	0.788198	2.638612
H	-1.332036	-1.591059	2.144235
H	-0.624343	-3.090811	-0.452320
H	-4.141220	2.352686	1.516210

TS$_{5-2}$

C	-2.948641	1.535775	-0.666212
C	-4.156305	1.601063	0.018556
C	-4.573333	0.568081	0.878246
C	-3.796955	-0.566894	1.083390
C	-2.585802	-0.631943	0.395309
C	-2.168149	0.397993	-0.462863
H	-2.624304	2.334851	-1.328746
H	-4.795447	2.472216	-0.111291
H	-5.526526	0.660746	1.395201
H	-4.121296	-1.363633	1.749448
N	-1.569498	-1.585863	0.348200
C	-0.575363	-1.189628	-0.476283
N	-0.939154	-0.004912	-0.961911
H	-0.246493	0.451614	-1.572645
H	-1.535878	-2.440761	0.883440
C	1.178207	-1.602994	-1.030507
N	1.886009	-1.822646	0.196759
C	2.568995	-0.601416	0.418614
C	3.337018	-0.182384	1.489508
C	2.322843	0.211412	-0.713993
C	3.889425	1.106538	1.430839
H	3.509166	-0.828366	2.349847
C	2.866869	1.499090	-0.751338
N	1.566380	-0.456745	-1.663995
C	3.648846	1.928306	0.326815
H	4.502794	1.468493	2.253969
H	2.700836	2.140348	-1.615773
H	0.971573	-2.495047	-1.621603
H	4.082494	2.927729	0.304122
H	2.430987	-2.675020	0.240183

TS$_{10\text{-}NHC}$

C	4.165091	-1.257452	-0.154962
C	5.214371	-0.352214	-0.341697
C	5.018853	1.026041	-0.203215
C	3.764591	1.551329	0.123678
C	2.721298	0.651261	0.303165
C	2.918381	-0.734591	0.168747
H	4.322341	-2.329656	-0.257160
H	6.202910	-0.728662	-0.598445
H	5.857869	1.703195	-0.352454
H	3.615781	2.624007	0.234251
N	1.385612	0.802070	0.652851
C	0.685817	-0.391658	0.670718
N	1.694234	-1.316671	0.463617
H	1.508222	-2.303586	0.390980
H	0.947358	1.680551	0.878688
C	-0.685817	-0.391651	-0.670725
N	-1.385612	0.802077	-0.652845
C	-2.721298	0.651264	-0.303159
C	-3.764590	1.551330	-0.123662
C	-2.918381	-0.734589	-0.168754
C	-5.018853	1.026039	0.203228
H	-3.615781	2.624009	-0.234224
C	-4.165091	-1.257454	0.154951
N	-1.694234	-1.316667	-0.463632
C	-5.214370	-0.352217	0.341697
H	-5.857868	1.703192	0.352475
H	-4.322341	-2.329658	0.257139
H	-6.202909	-0.728668	0.598442
H	-0.947359	1.680559	-0.878676
H	-1.508222	-2.303583	-0.391005

TS$_{6-11}$

C	4.165984	1.302291	0.490894
C	5.358283	0.750801	0.001897
C	5.379912	-0.520249	-0.576964
C	4.218684	-1.292060	-0.695855
C	3.034281	-0.752766	-0.212828
C	3.027411	0.533081	0.356887
H	4.142878	2.293834	0.938440

H	6.278931	1.327003	0.069376
H	6.323947	-0.919220	-0.944205
H	4.230894	-2.284146	-1.141056
N	1.738844	-1.316502	-0.239440
C	0.948513	-0.486028	0.326059
N	1.676385	0.769487	0.784548
H	1.571111	0.925398	1.790701
H	0.713983	1.264521	0.275543
C	-0.437111	0.032725	0.183646
N	-1.429652	-0.765906	0.317337
C	-2.768756	-0.394612	0.134242
C	-3.700526	-1.416500	0.385711
C	-3.248727	0.874442	-0.290858
C	-5.065534	-1.230162	0.233121
H	-3.294071	-2.374943	0.704827
C	-4.637067	1.049157	-0.449488
N	-2.401432	1.934988	-0.510846
C	-5.529170	0.019780	-0.193581
H	-5.761368	-2.041532	0.435459
H	-5.003872	2.022935	-0.775961
H	-2.758400	2.697002	-1.067070
H	-1.413427	1.723070	-0.577729
H	-6.596681	0.192217	-0.326804

$TS_{11\text{-}12}$

C	-3.420122	-0.632937	1.178486
C	-4.356606	0.382070	1.048371
C	-4.188007	1.425796	0.113814
C	-3.078594	1.485368	-0.718114
C	-2.138764	0.460023	-0.581580
C	-2.292431	-0.592510	0.348104
H	-3.547288	-1.440290	1.896821
H	-5.244293	0.377557	1.678617
H	-4.947147	2.203062	0.044382
H	-2.950625	2.291683	-1.437459
N	-0.944622	0.192004	-1.216753
C	-0.447926	-0.952947	-0.669858
N	-1.213388	-1.460963	0.266095
H	0.755604	-1.902137	1.116630
H	-0.455932	0.725314	-1.923483
C	0.849439	-1.520276	-1.194202
N	1.577261	-1.821021	0.515874
C	2.262643	-0.545948	0.554182
C	2.930691	-0.015682	1.646027
C	2.279578	0.075417	-0.727019
C	3.590172	1.207563	1.504447
H	2.923982	-0.533840	2.604718
C	2.954076	1.303063	-0.847623
N	1.745419	-0.687721	-1.733072
C	3.589936	1.851623	0.261680
H	4.094882	1.656554	2.356839
H	2.981997	1.800911	-1.814870
H	0.723236	-2.508439	-1.651811
H	4.105823	2.805855	0.157766
H	2.202559	-2.616057	0.646552

TSa

C	-0.925819	1.429451	-0.013491
C	-2.108032	0.701613	0.003031
C	-2.115214	-0.708847	0.015542
C	-0.930718	-1.431458	0.005762
C	0.265802	-0.708277	-0.003070
C	0.264204	0.693472	0.003549
H	-0.931150	2.517668	-0.024607
H	-3.057914	1.234242	0.007917
H	-3.068808	-1.233401	0.025882
H	-0.920477	-2.519570	-0.002780
N	1.593978	-1.153028	-0.091511
C	2.412699	-0.037570	-0.071913
N	1.597137	1.060378	0.022471
H	1.947053	2.005771	0.058567
H	2.515968	-0.986461	0.781842

TSb

C	-4.778777	1.074886	0.000094
C	-5.566197	-0.072938	0.000017
C	-4.987088	-1.355400	-0.000073
C	-3.606430	-1.526304	-0.000088
C	-2.803370	-0.381075	-0.000011
C	-3.394788	0.896701	0.000079
H	-5.229076	2.066427	0.000163
H	-6.650853	0.022653	0.000025
H	-5.636169	-2.229878	-0.000131
H	-3.161436	-2.519976	-0.000157
N	-1.427109	-0.216775	-0.000002
C	-1.124917	1.102582	0.000089
N	-2.324479	1.774292	0.000138
H	-2.396261	2.779115	0.000211
H	-0.253855	-0.940811	-0.000062
H	0.645146	1.081015	0.000082
N	1.470801	0.365971	0.000025
C	2.854387	0.435943	0.000021
C	1.063137	-0.908143	-0.000068
C	3.745395	1.511484	0.000096
C	3.318699	-0.889710	-0.000083
N	2.174570	-1.681175	-0.000135
C	5.101424	1.210681	0.000063
H	3.387953	2.538590	0.000177
C	4.680231	-1.194186	-0.000117
H	2.155820	-2.689482	-0.000213
C	5.562057	-0.120006	-0.000042
H	5.827504	2.021505	0.000119
H	5.038617	-2.221638	-0.000198
H	6.633550	-0.310799	-0.000065

RC (X=O)

C	-5.289044	0.158547	0.000316
C	-5.264483	-1.231814	0.000294
C	-4.053495	-1.930600	0.000064
C	-2.853416	-1.231226	-0.000144
C	-2.880006	0.164705	-0.000122
C	-4.091296	0.873619	0.000107
H	-6.223762	0.714832	0.000495
H	-6.205656	-1.779377	0.000459
H	-4.045660	-3.018033	0.000048
H	-1.888336	-1.734500	-0.000324
N	-1.700733	0.902622	-0.000324
C	-0.732073	1.566311	-0.000481
H	-3.243132	2.586864	-0.000024
C	0.732556	-1.567313	-0.000397
N	1.701038	-0.903359	-0.000322
C	2.880050	-0.165011	-0.000115
C	2.852830	1.230912	-0.000118
C	4.091625	-0.873419	0.000101
C	4.052565	1.930840	0.000093
H	1.887364	1.733436	-0.000280
C	5.289048	-0.157787	0.000309
C	5.263860	1.232560	0.000301
H	4.044271	3.018268	0.000094
H	6.224032	-0.713625	0.000474
H	3.244214	-2.587022	-0.000057
H	6.204796	1.780531	0.000467
O	-4.139417	2.225042	0.000134
O	4.140343	-2.224812	0.000107

TS$_{RC-1}$ (X=O)

C	-5.276439	-0.246569	-0.000035
C	-5.305234	1.143493	-0.000017
C	-4.121484	1.890186	0.000012
C	-2.896294	1.235771	0.000023
C	-2.864401	-0.159243	0.000003
C	-4.048288	-0.910350	-0.000026
H	-6.189317	-0.838024	-0.000055
H	-6.266679	1.654942	-0.000022
H	-4.158284	2.977291	0.000027
H	-1.954033	1.781641	0.000052
N	-1.654809	-0.870788	0.000021
C	-0.458188	-0.745503	0.000054
H	-3.108126	-2.555760	-0.000110
C	0.458215	0.745613	0.000060
N	1.654843	0.870871	0.000047
C	2.864402	0.159266	0.000021
C	2.896240	-1.235748	0.000042
C	4.048318	0.910330	-0.000025
C	4.121404	-1.890211	0.000018
H	1.953959	-1.781582	0.000077
C	5.276444	0.246500	-0.000046
C	5.305183	-1.143562	-0.000027
H	4.158163	-2.977317	0.000031
H	6.189346	0.837918	-0.000079
H	3.108207	2.555736	0.000066
H	6.266608	-1.655048	-0.000043
O	-4.030326	-2.261843	-0.000035
O	4.030408	2.261816	-0.000060

INT1 (X=O)

C	-5.344417	0.358457	0.000198
C	-5.468968	-1.024770	0.000065
C	-4.336890	-1.853587	-0.000125
C	-3.073063	-1.285825	-0.000168
C	-2.928881	0.109314	-0.000043
C	-4.071207	0.934046	0.000145
H	-6.214141	1.011800	0.000344
H	-6.462938	-1.470000	0.000104
H	-4.451112	-2.935438	-0.000242
H	-2.177079	-1.906183	-0.000326
N	-1.705455	0.797508	-0.000147
C	-0.604816	0.199765	-0.000323
H	-3.007664	2.483702	0.000243
C	0.604809	-0.199685	-0.000215
N	1.705436	-0.797457	-0.000125
C	2.928877	-0.109294	-0.000056
C	3.073107	1.285842	-0.000196
C	4.071183	-0.934059	0.000137
C	4.336951	1.853566	-0.000131
H	2.177142	1.906227	-0.000362
C	5.344410	-0.358510	0.000213
C	5.469003	1.024714	0.000078
H	4.451209	2.935413	-0.000248
H	6.214115	-1.011878	0.000371
H	3.007587	-2.483683	0.000137
H	6.462986	1.469915	0.000134
O	-3.955838	2.276669	0.000274
O	3.955768	-2.276678	0.000260

RC (X=NCO_2Me)

C	4.877666	-2.110936	-0.000056
C	4.236974	-3.347464	0.000008
C	2.845834	-3.449195	0.000079
C	2.076814	-2.291753	0.000086
C	2.713596	-1.052517	0.000022
C	4.118050	-0.934594	-0.000050
H	5.959341	-2.044727	-0.000111
H	4.845683	-4.250216	0.000002
H	2.361059	-4.422776	0.000129
H	0.988772	-2.317003	0.000139
N	1.950961	0.111568	0.000029
C	1.329570	1.107531	0.000031
N	4.659133	0.351802	-0.000112
H	4.011416	1.129446	-0.000099
C	-1.327988	-1.104490	0.000193
N	-1.950486	-0.109223	0.000185
C	-2.714282	1.054095	0.000086
C	-2.078572	2.293894	-0.000022
C	-4.118602	0.934850	0.000107
C	-2.848517	3.450675	-0.000113
H	-0.990560	2.319560	-0.000033
C	-4.879213	2.110578	0.000013
N	-4.658483	-0.352026	0.000228
C	-4.239586	3.347648	-0.000095
H	-2.364646	4.424704	-0.000199
H	-5.960830	2.043494	0.000027
H	-4.010028	-1.129055	0.000283
H	-4.849101	4.249858	-0.000167
C	5.988376	0.690515	-0.000183
O	6.929910	-0.068515	-0.000133
O	6.095879	2.034625	-0.000101
C	7.441943	2.517176	-0.000058
H	7.969326	2.166396	-0.893057
H	7.360355	3.605387	0.000022
H	7.969319	2.166265	0.892893
C	-5.987422	-0.692020	0.000262
O	-6.929679	0.066105	-0.000015
O	-6.093619	-2.036216	0.000063
C	-7.439220	-2.520074	-0.000253
H	-7.966769	-2.169626	-0.893283
H	-7.356571	-3.608205	-0.000378
H	-7.967099	-2.169853	0.892671

TS$_{RC-1}$ (X=NCO_2Me)

C	-4.852032	2.096664	0.000014
C	-4.246503	3.350438	-0.000008
C	-2.857320	3.491060	-0.000058
C	-2.058692	2.353828	-0.000083
C	-2.655059	1.095667	-0.000058
C	-4.055086	0.944492	-0.000015
H	-5.931479	1.999616	0.000045
H	-4.879382	4.236534	0.000012
H	-2.401645	4.478848	-0.000081
H	-0.971555	2.415697	-0.000120
N	-1.867336	-0.067276	-0.000088
C	-0.732237	-0.466529	-0.000091
N	-4.553714	-0.358312	0.000012
H	-3.867022	-1.102274	0.000007
C	0.732218	0.466457	0.000011
N	1.867321	0.067222	0.000043
C	2.655071	-1.095705	0.000011
C	2.058735	-2.353880	-0.000030
C	4.055094	-0.944493	0.000028
C	2.857392	-3.491091	-0.000053
H	0.971599	-2.415773	-0.000044
C	4.852070	-2.096645	0.000001
N	4.553686	0.358319	0.000078
C	4.246571	-3.350434	-0.000041
H	2.401742	-4.478891	-0.000083
H	5.931514	-1.999568	0.000016
H	3.866981	1.102268	0.000078
H	4.879472	-4.236515	-0.000059
C	-5.867396	-0.744404	0.000041

O	-6.836900	-0.020285	0.000069
O	-5.928697	-2.092829	0.000028
C	-7.257348	-2.620042	0.000052
H	-7.796851	-2.287362	-0.892706
H	-7.139836	-3.705074	0.000085
H	-7.796842	-2.287306	0.892794
C	5.867367	0.744437	0.000022
O	6.836882	0.020335	-0.000026
O	5.928647	2.092865	0.000019
C	7.257290	2.620100	0.000059
H	7.796821	2.287395	-0.892673
H	7.139759	3.705130	0.000051
H	7.796767	2.287407	0.892827

INT1 (X=NCO_2Me)

C	-4.947950	2.057582	0.000004
C	-4.416025	3.342538	0.000115
C	-3.034020	3.561766	0.000314
C	-2.176532	2.473264	0.000402
C	-2.687261	1.169406	0.000297
C	-4.085371	0.952131	0.000092
H	-6.020130	1.898883	-0.000147
H	-5.097418	4.191991	0.000049
H	-2.636209	4.574397	0.000404
H	-1.095436	2.609158	0.000568
N	-1.882155	0.016278	0.000406
C	-0.631785	0.082402	0.000595
N	-4.515112	-0.372433	0.000007
H	-3.780554	-1.070366	0.000071
C	0.631796	-0.082293	0.000690
N	1.882169	-0.016206	0.000508
C	2.687241	-1.169359	0.000363
C	2.176474	-2.473203	0.000475
C	4.085357	-0.952124	0.000101
C	3.033931	-3.561729	0.000334
H	1.095375	-2.609065	0.000690
C	4.947904	-2.057600	-0.000034
N	4.515135	0.372427	0.000006
C	4.415943	-3.342542	0.000082
H	2.636092	-4.574349	0.000424
H	6.020089	-1.898932	-0.000228
H	3.780598	1.070381	0.000103
H	5.097311	-4.192014	-0.000023
C	-5.805096	-0.829445	-0.000206
O	-6.814337	-0.160957	-0.000445
O	-5.792669	-2.179576	-0.000405
C	-7.090059	-2.778726	-0.000802
H	-7.647091	-2.476160	-0.893586
H	-6.913399	-3.855770	-0.000871
H	-7.647551	-2.476344	0.891754
C	5.805133	0.829401	-0.000259
O	6.814353	0.160882	-0.000499
O	5.792746	2.179533	-0.000387
C	7.090154	2.778644	-0.000768
H	7.647166	2.476107	-0.893573
H	6.913525	3.855693	-0.000779
H	7.647647	2.476200	0.891767

2. Bibliography

(1) Zhao, Y.; Truhlar, D. G. Density Functionals with Broad Applicability in Chemistry. *Acc. Chem. Res.* **2008**, *41* (2), 157–167. https://doi.org/10.1021/ar700111a

(2) Papajak, E.; Zheng, J.; Xu, X.; Leverentz, H. R.; Truhlar, D. G. Perspectives on Basis Sets Beautiful: Seasonal Plantings of Diffuse Basis Functions. *J. Chem. Theory Comput.* **2011**, *7* (10), 3027–3034. https://doi.org/10.1021/ct200106a

(3) a) Zhang, Z.; Su, M. Reactivity and Activation Barrier Origins of Heavy Isocyanide-Like Molecules Featuring a Group 14═P Double Bond Toward Methyl Iodide: A Chemical Insight. *Chem. Asian J.* **2025**, e70209. https://doi.org/10.1002/asia.70209. b) Wang, Y.; Wei, D.; Zhu, Y.; Liu, P.; Tang, M. A DFT Study on the Reaction Mechanisms of Isocyanide-Based Multicomponent Synthesis of Polysubstituted Cyclopentenes. *Comp. Theor. Chem.* 2013, 1018, 85–90. https://doi.org/10.1016/j.comptc.2013.06.018

(4) Bauernschmitt, R.; Häser, M.; Treutler, O.; Ahlrichs, R. Calculation of Excitation Energies within Time-Dependent Density Functional Theory Using Auxiliary Basis Set Expansions. *Chem. Phys. Lett.* **1997**, *264* (6), 573–578. https://doi.org/10.1016/S0009-2614(96)01343-7

(5) (1) Pastor, A.; Lopez-Leonardo, C.; Cutillas-Font, G.; Martinez-Cuezva, A.; Marin-Luna, M.; Garcia-Lopez, J.-A.; Saura-Llamas, I.; Alajarin, M. Unveiling the Phosphine-Mediated N -Transfer from Azide to Isocyanide En Route to Carbodiimides and 4-Imino-1,3,2-Diazaphosphetidines. *Org. Lett.* **2025**, 27 (1), 73–79. https://doi.org/10.1021/acs.orglett.4c03902

(6) M. J. Frisch, G. W. Trucks, H. B. Schlegel, G. E. Scuseria, M. A. Robb, J. R. Cheeseman, G. Scalmani, V. Barone, G. A. Petersson, H. Nakatsuji, X. Li, M. Caricato, A. V. Marenich, J. Bloino, B. G. Janesko, R. Gomperts, B. Mennucci, H. P. Hratchian, J. V. Ortiz, A. F. Izmaylov, J. L. Sonnenberg, D. Williams-Young, F. Ding, F. Lipparini, F. Egidi, J. Goings, B. Peng, A. Petrone, T. Henderson, D. Ranasinghe, V. G. Zakrzewski, J. Gao, N. Rega, G. Zheng, W. Liang, M. Hada, M. Ehara, K. Toyota, R. Fukuda, J. Hasegawa, M. Ishida, T. Nakajima, Y. Honda, O. Kitao, H. Nakai, T. Vreven, K. Throssell, J. A. Montgomery, Jr., J. E. Peralta, F. Ogliaro, M. J. Bearpark, J. J. Heyd, E. N. Brothers, K. N. Kudin, V. N. Staroverov, T. A. Keith, R. Kobayashi, J. Normand, K. Raghavachari, A. P. Rendell, J. C. Burant, S. S. Iyengar, J. Tomasi, M. Cossi, J. M. Millam, M. Klene, C. Adamo, R. Cammi, J. W. Ochterski, R. L. Martin, K. Morokuma, O. Farkas, J. B. Foresman, and D. J. Fox,. *Gaussian 16, Revision C.01*; Gaussian, Inc.: Wallingford CT, 2016.

(7) CYLview20; C. Y. Legault, Université de Sherbrooke, 2020 (http://www.cylview.org)
